# Computational fluid dynamics for enhanced tracheal bioreactor design and long-segment graft recellularization

**DOI:** 10.1038/s41598-020-80841-w

**Published:** 2021-01-13

**Authors:** Hankyu Lee, Alba E. Marin-Araujo, Fabio G. Aoki, Siba Haykal, Thomas K. Waddell, Cristina H. Amon, David A. Romero, Golnaz Karoubi

**Affiliations:** 1 Department of Mechanical and Industrial Engineering, University of Toronto, 5 King’s College Road, Toronto, ON M5S 3G8 Canada; 2 Institute of Biomaterials and Biomedical Engineering, University of Toronto, 164 College Street, Toronto, ON M5S 3G9 Canada; 3 Latner Research Laboratories, Division of Thoracic Surgery, University Health Network, 101 College Street, Toronto, ON M5G 1L7 Canada; 4 Institute of Science and Technology, Federal University of Sao Paulo, R. Talim, 330, Sao Jose dos Campos, SP 12231-280 Brazil; 5 Division of Plastic & Reconstructive Surgery, University Health Network, University of Toronto, 200 Elizabeth Street, Toronto, ON M5G2C4 Canada

**Keywords:** Respiratory system models, Computational models, Tissue engineering, Biomedical engineering

## Abstract

Successful re-epithelialization of de-epithelialized tracheal scaffolds remains a challenge for tracheal graft success. Currently, the lack of understanding of the bioreactor hydrodynamic environment, and its relation to cell seeding outcomes, serve as major obstacles to obtaining viable tracheal grafts. In this work, we used computational fluid dynamics to (a) re-design the fluid delivery system of a trachea bioreactor to promote a spatially uniform hydrodynamic environment, and (b) improve the perfusion cell seeding protocol to promote homogeneous cell deposition. Lagrangian particle-tracking simulations showed that low rates of rotation provide more uniform circumferential and longitudinal patterns of cell deposition, while higher rates of rotation only improve circumferential uniformity but bias cell deposition proximally. Validation experiments with human bronchial epithelial cells confirm that the model accurately predicts cell deposition in low shear stress environments. We used the acquired knowledge from our particle tracking model, as a guide for long-term tracheal repopulation studies. Cell repopulation using conditions resulting in low wall shear stress enabled enhanced re-epithelialization of long segment tracheal grafts. While our work focuses on tracheal regeneration, lessons learned in this study, can be applied to culturing of any tissue engineered tubular scaffold.

## Introduction

Tracheal injury, stenosis, and malignancy demand surgical management or tracheal reconstruction, however, the latter is an unmet clinical need as tracheal grafts and transplants fail due to the lack of a functioning epithelium, immune rejection, and graft ischemia^1^. Repair and replacement of trachea has been evaluated and included assessment of autologous^2^ and nonviable tissues^3^, foreign materials^4^, and naturally derived biological acellular scaffolds^5^. For long-segment tracheal defects, tissue-engineered tracheal replacements have shown potential as a therapeutic alternative^6–9^. Generation of biological tracheal replacements involve the re-population of fully or partially decellularized tracheal scaffolds inside a bioreactor system before in vivo implantation. However, successful epithelialization of tracheal scaffolds remains a challenge. We have previously demonstrated the use of a double-perfusion bioreactor system for recellularization of acellular porcine tracheal grafts^10^ and repopulation of partially decellularized (de-epithelialized) tracheal grafts^11^. Our objective for this study was to enhance our previously developed bioreactor system^10^, by improving upon design limitations such as a lack of parameter control and monitoring (temperature, flow rate, rotational speed, etc.), and by re-designing its flow delivery subsystem to achieve a homogeneous hydrodynamic environment that would improve recellularization outcomes. We hypothesized that in silico modelling of bioreactor modifications would allow for a clear understanding of their effect on the hydrodynamic environment within the construct, providing in silico guidance to accelerate the optimization of re-epithelialization protocols for tracheal scaffolds.

Although recent attempts have been made, there is a lack of experimentally validated computational models for cell deposition, attachment and growth on bioreactor-cultured constructs; essential for successful recellularization. Computational Fluid Dynamics (CFD) has been used to quantify the flow velocity fields and shear stresses on tissue scaffolds^12–15^. These studies, though useful in understanding the hydrodynamic environment of the scaffolds, fail to consider the effect of the bioreactor design and the flow delivery mechanism^16–20^. From an engineering design perspective, the effect of the bioreactor design on its hydrodynamic environment is a significant and controllable variable that is under-appreciated in the literature. CFD has also been used to predict the cell deposition on scaffolds. Specifically, using CFD to track the cell trajectory and the cell deposition distribution on small sections of synthetic porous scaffolds^20–25^.

Repopulating a fully functional pseudostratified epithelial lining onto a de-epithelialized graft is a major challenge that is not unique to porous scaffolds. Without a fully functional epithelium, tracheal stenosis, collapse, fibrosis and infections occur after transplantation^9,10^. We have found no previous work focused on predicting cell deposition patterns in tubular scaffolds such as the trachea. In this work, CFD is, first, used as a tool for designing the bioreactor to create an improved mechanical environment for re-epithelialization of tracheal grafts. Secondly, CFD, together with the Discrete Phase Model (DPM) was employed to predict the cell deposition patterns on a tubular scaffold inside our bioreactor. This particle tracking model was experimentally validated using human bronchial epithelial cells (BEAS-2B) cells in the trachea bioreactor. Finally, we used the acquired knowledge from our particle tracking model, as a guide for long-term tracheal repopulation studies.

## Results

### Enhanced bioreactor design

**Figure 1:**
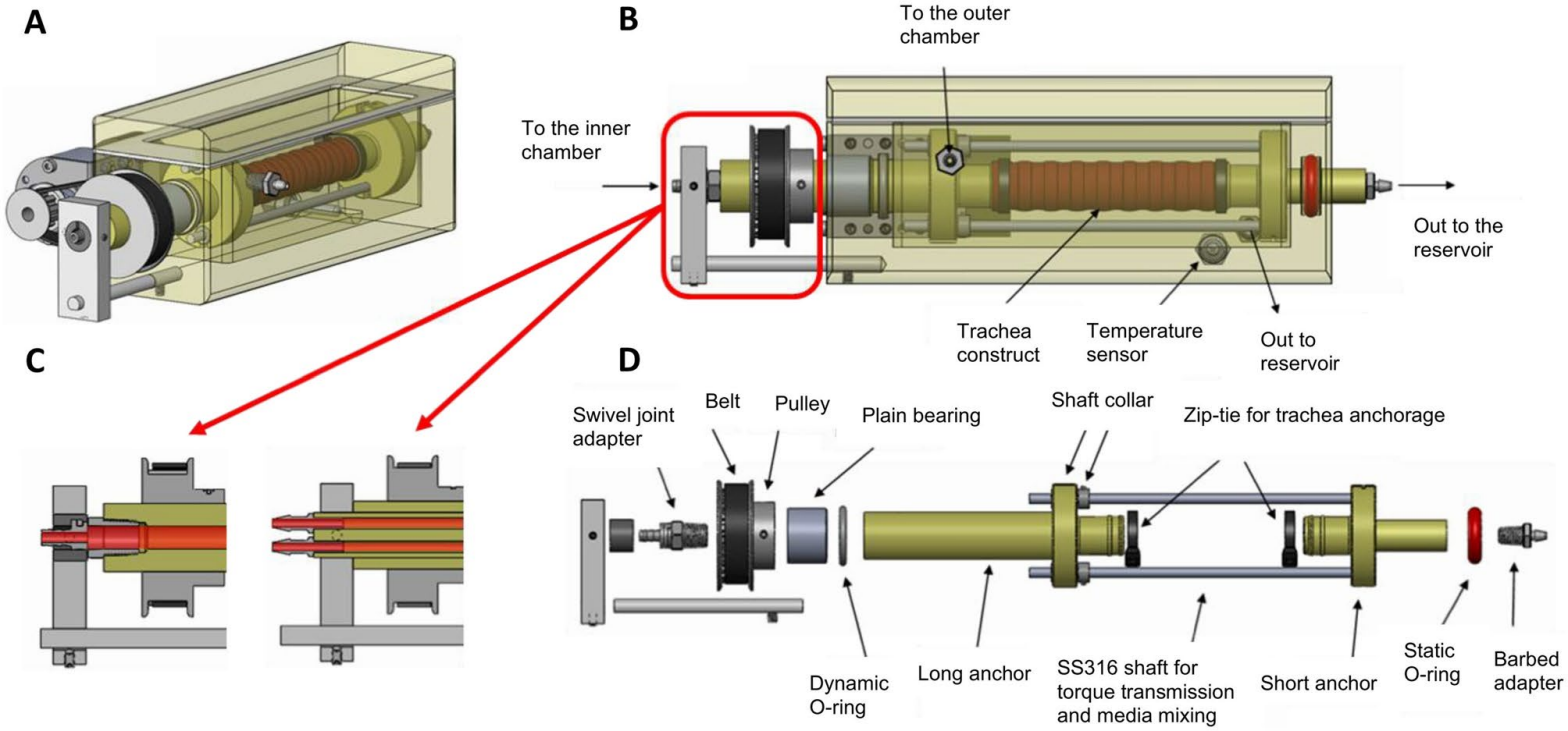
CAD model of the double-chamber tubular-scaffold bioreactor. The bioreactor body and lid are shown as translucent. (**A**) An isometric view of the bioreactor showing the motor, pulleys and belt which allows the tracheal rotation; (**B**) Side view of the trachea bioreactor; (**C**) Magnified cross-sectional view of two inlet designs; (**D**) Exploded view of the bioreactor components without the body and the lid. Figure was generated using Solidworks v2016 (Dassault Systemes, Vélizy-Villacoublay, France; https://www.solidworks.com).

The new bioreactor system is shown schematically in Fig. 1. Figure 1A,B show the trachea bioreactor assembly with the trachea securely tightened between two rotating anchors. The tracheal graft wall isolates the interior lumen chamber from the exterior culture chamber which allows for different microenvironment requirements of the pseudostratified ciliated epithelium compared to the chondrocytes. Figure 1C shows a cross section of the single rotating inlet and the double static inlet that the bioreactor platform is compatible with. The double static inlet design was intended to allow injection of liquid and air separately into the bioreactor. The injection of both air and liquid is also possible with the single inlet design, with the difference being the need for pre-mixing of air and liquid flows prior to injection into the bioreactor (single) inlet. In any case, this modular design enables control over the flow field of the inner chamber by tailoring the inlet to the desired experimental conditions. The electronic control unit includes actuators, sensors, microcontrollers and a laptop. The DC motor (Fig. 1A) allows tracheal rotation along its longitudinal axis from 0 to 30 rpm. The rotation allows circulation and mixing of micronutrients, provides control of the lumen wall shear stress, positional control during perfusion cell seeding and a periodic exposure to both air and liquid when an air–liquid-interface is achieved in the inner chamber. Microcontrollers control the rotational velocity of the trachea and the flow rate of the custom peristaltic pumps. An inline flowmeter, and a stainless-steel housed thermistor (Figs. 1, S1) enable data acquisition and real time. A sampling valve located at the lumen chamber outlet allows for sample fluid collection via a syringe for subsequent analysis (i.e. for dissolved O_2_, CO_2_, glucose, metabolite concentrations and pH) (Fig. S2).

### Wall shear stress

**Figure 2:**
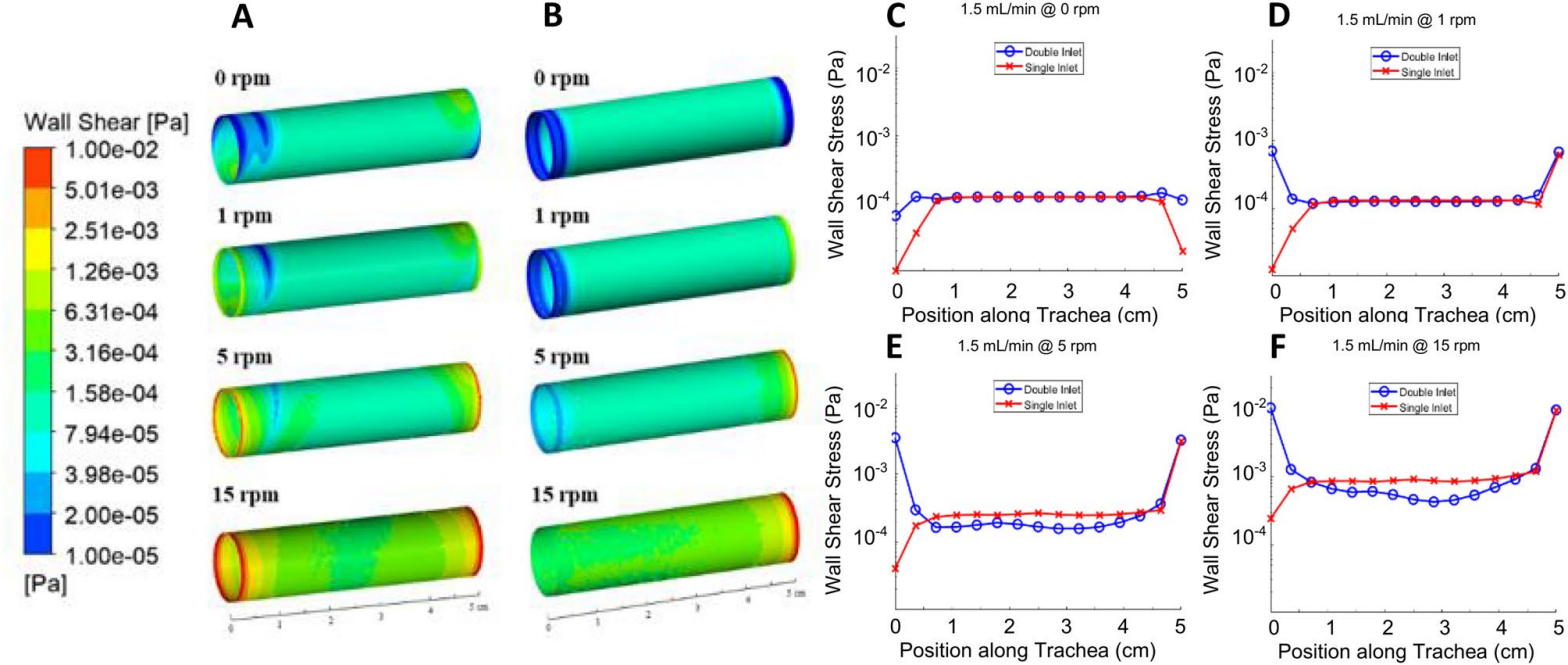
Luminal wall shear stress within the bioreactor. Wall shear stress contours with the (**A**) double (blue) and (**B**) single (red) inlet designs at different tracheal rotational speeds; comparison plots of the lumen wall shear stress at 1.5 mL/min at (**C**) 0 rpm, (**D**) 1 rpm, (**E**) 5 rpm, and (**F**) 15 rpm. (A) and (**B**) were generated using ANSYS Workbench v19.1 (ANSYS Inc., Cannonsburgh, PA, USA; https://www.ansys.com). (**B**), (**C**) and (**D**) were generated using MATLAB 2018b (Mathworks Inc., Natick, Massachusetts, USA; https://www.mathworks.com/).

The in silico computed shear stress distribution in the inner surface of the lumen wall at different tracheal rotation speeds for the double-inlet and single-inlet bioreactor designs are shown in Fig. 2A,B respectively. For the double-inlet design (Fig. 2A), it can be observed that the rotation rate has a significant effect on the minimum, maximum and average shear stress values (Fig. 2C–F), which increase several orders of magnitude, from 0.00063 to 0.025 Pa, as the rotation speed is increased from 0 rpm (no rotation) to 15 rpm. A similar behavior is observed for the single-inlet design (Fig. 2B), for which the average shear stress increases from 0.00012 at 0 rpm to 0.0015 Pa at 15 rpm.

The results shown in Fig. 2C–F also show that the single-inlet design (red lines with cross markers in the figures) results in a more uniform shear stress profile, as evidenced by curves with fewer changes in slope and smaller departures from the average value at the trachea inlet. This is particularly apparent in the double static inlet platform. For example, at 15 rpm, the vicinity of the inlet and outlet exhibit significantly larger shear stress values (approx. 0.01 Pa), seen in the figures are red ring-like structures, compared to an average of 0.001 Pa across the rest of the lumen surface. Note also that the shear stress profiles in the trachea outlet are similar in both the double-inlet and single-inlet bioreactors, since no changes were made to the design in the outlet region, which consists of a stationary short anchor around which the trachea rotates, thus creating a strong velocity gradient (and thus shear stress gradient) in this region.

In other experiments we show that, for a fixed rotation speed, there is minimal effect of different flow rates on the wall shear stress distributions. We have simulated flow rates ranging from 1.5 to 12 mL/min at rotation speeds of 0, 5, and 20 rpm. As an example, for a 5 rpm rotation rate, increasing the flow rate from 1.5 mL/min to 3, 6, and 12 mL/min increases the wall shear stress from 0.0003 Pa to 0.00045, 0.00082 and 0.0018 Pa. Fig. S3 shows the effect of flow rate on the shear stress distribution in the inner surface of the lumen wall at a rotation speed of 5 rpm for the single inlet bioreactor design. The magnitude of this increase, however, is significantly lower than what can be achieved by increases in the rotation rate.

### Particle deposition density

**Figure 3:**
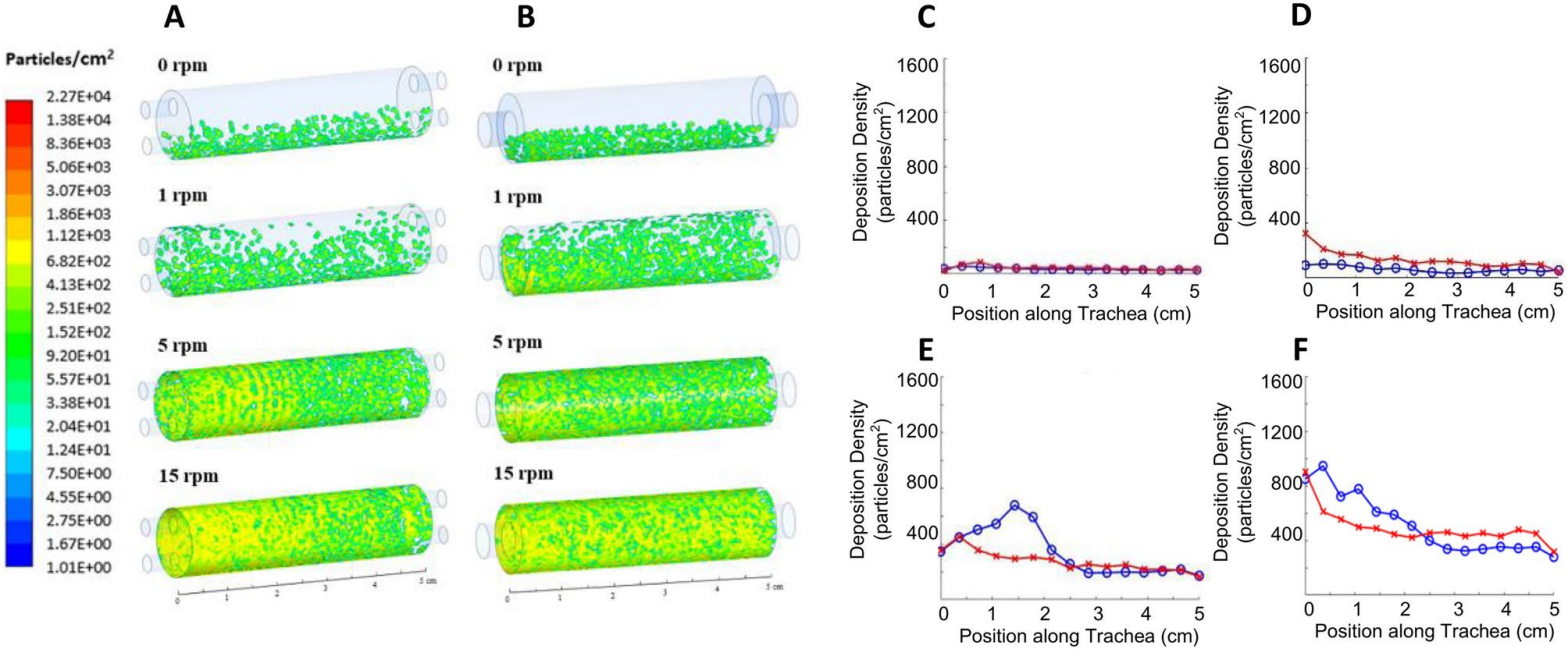
Particle deposition density. Particle deposition density contours of the double (**A**) and single (**B**) inlet designs. Particle deposition density plots at 1.5 mL/min of the double (blue) and single (red) inlet designs at different rotational speeds: 0 rpm (**C**), 1 rpm (**D**), 5 rpm (**E**), and 15 rpm (**F**). (**A**) and (**B**) were generated using ANSYS Workbench v19.1 (ANSYS Inc., Cannonsburgh, PA, USA; https://www.ansys.com). (**B**,**C**–**F**) were generated using MATLAB 2018b (Mathworks Inc., Natick, Massachusetts, USA; https://www.mathworks.com/).

In the absence of rotation (0 rpm case), gravity causes the particles to deposit preferentially towards the bottom of the tracheal scaffold, although sporadically. The remaining cells are carried by the flow out of the fluid domain via the outlet, due to the relative magnitude of the drag and gravitational forces acting on the cells. The velocity streamlines (Fig. S4) show the potential trajectories of some of these particles. Introduction of rotation (e.g. 5 rpm) allows for a more relatively uniform particle deposition along the circumferential direction. Increasing the rotational velocity further causes a greater number of particles to deposit near the inlet and prevents many of injected particles to travel the entire length of the trachea (15 rpm). The helical structure of the flow field (Figs. 3, S4) exerts a centrifugal force on the particles that is proportional to the rotational velocity. This centrifugal force causes the cells to deposit onto the lumen at a higher density than in the absence of rotation and to preferentially deposit towards the inlet at higher rotational velocities.

The two different inlet designs that were investigated exhibited different cell deposition patterns. For example, at 1 rpm, the single rotating inlet design has a greater circumferential uniformity in cell deposition compared with the double static inlet design. At 5 rpm and 15 rpm, the single rotating inlet design exhibits more uniform cell accretion density magnitudes along the length of the trachea, from the inlet to the outlet (Fig. 3). Overall, the particle deposition results were observed to be very sensitive to the bioreactor inlet and outlet designs. Figure 3 also shows the circumferentially averaged cell deposition density as a function of longitudinal position along the trachea, from the inlet (0 cm) to the outlet (5 cm), for several rotation speeds. For 5 and 15 rpm, the double inlet design results in higher particle deposition rates near the inlet. This is likely a result of differences in the location of the inlet flow, with respect to the center axis of the tubular scaffold for the double inlet design. Specifically, the particles entering the tubular scaffold are positioned closer to the rotating wall which exposes these particles to a larger centrifugal force and a shorter travel distance to the wall. At both 5 and 15 rpm, the single inlet design shows a more uniform accretion density along the entire length of the trachea compared to the double inlet design (blue) which exhibits an initial peak average cell accretion density. Overall, these findings suggest that, by controlling the relative effect of two experimental parameters (rotation rate and flow rate), and by carefully designing the inlet design, the helicoidal structures of the flow can be controlled to achieve more uniform shear stress distributions and cell deposition patterns, both circumferentially and axially. Based on these findings, we selected the single-inlet bioreactor for all subsequent experiments and analysis discussed in the remainder of this manuscript.

### Experimental validation of the discrete phase model

**Figure 4:**
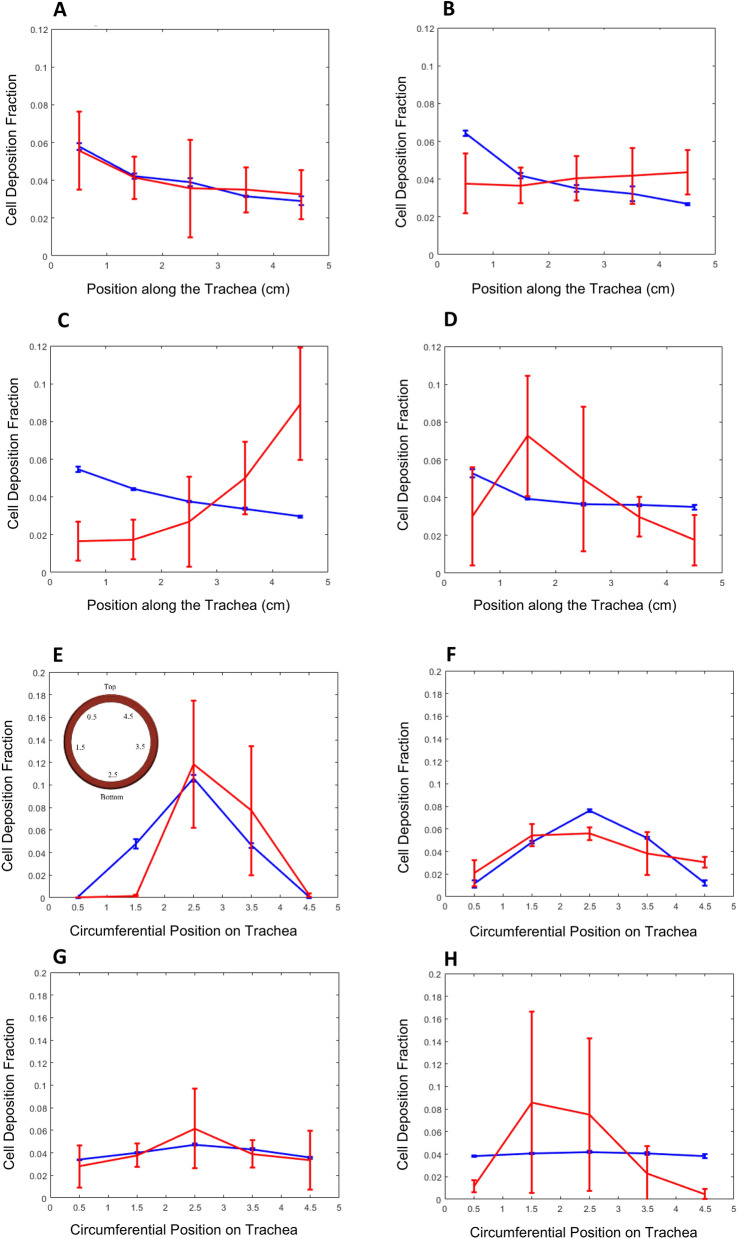
Averaged cell deposition density fraction plots along tracheal length and circumferential position. Simulation (blue) and experimental (red) results at 0 rpm (**A**,**E**), 1 rpm (**B**,**F**), 5 rpm (**C**,**G**), and 15 rpm (**D**,**H**), single rotating inlet bioreactor.

The in silico model was validated via biological experiments, by comparing the cell deposition density fraction in the single rotating inlet bioreactor obtained experimentally and extracted from the simulations. For this purpose, the cell deposition density fraction was defined as the number of cells deposited per unit area of scaffold surface, divided by the total number of cells that were deposited on the entire surface of the scaffold. Figure 4A–D were obtained by circumferentially averaging the cell deposition density fraction at every position along the length of the trachea. Figure 4E–H were obtained in a similar method by axially averaging the cell deposition density fraction at every position along the circumference of the trachea.

At 0 rpm, the general decreasing trend from the inlet to the outlet along the length of the trachea and the preferential cell deposition towards the bottom caused by gravity forces, observed in the validation experiments, is well captured by the simulations, providing strong evidence that the main physical phenomena are represented in the model. Inducing rotation, however, lead to discrepancies between the experimental and the simulation results. These discrepancies become more pronounced in the longitudinal direction and as the rotational velocity increases. At higher rotational velocities, the simulation results depart from the general trend of the experimental observations and show that the cell deposition density fraction increases from the inlet to the outlet and exhibits greater inter-sample variability. For instance, the 5 rpm results (Fig. 4C) show a higher cell deposition density fraction towards the outlet, and at 15 rpm the experimental results strongly deviate from the simulations. In contrast, experimental results for the circumferential distribution of cell deposition are well captured in the simulations at low (0 rpm, 1 rpm) and moderate (5 rpm) rotational speeds, with larger deviations and larger inter-sample variability at 15 rpm.

### In Silico guided cell attachment in bioreactor

**Figure 5:**
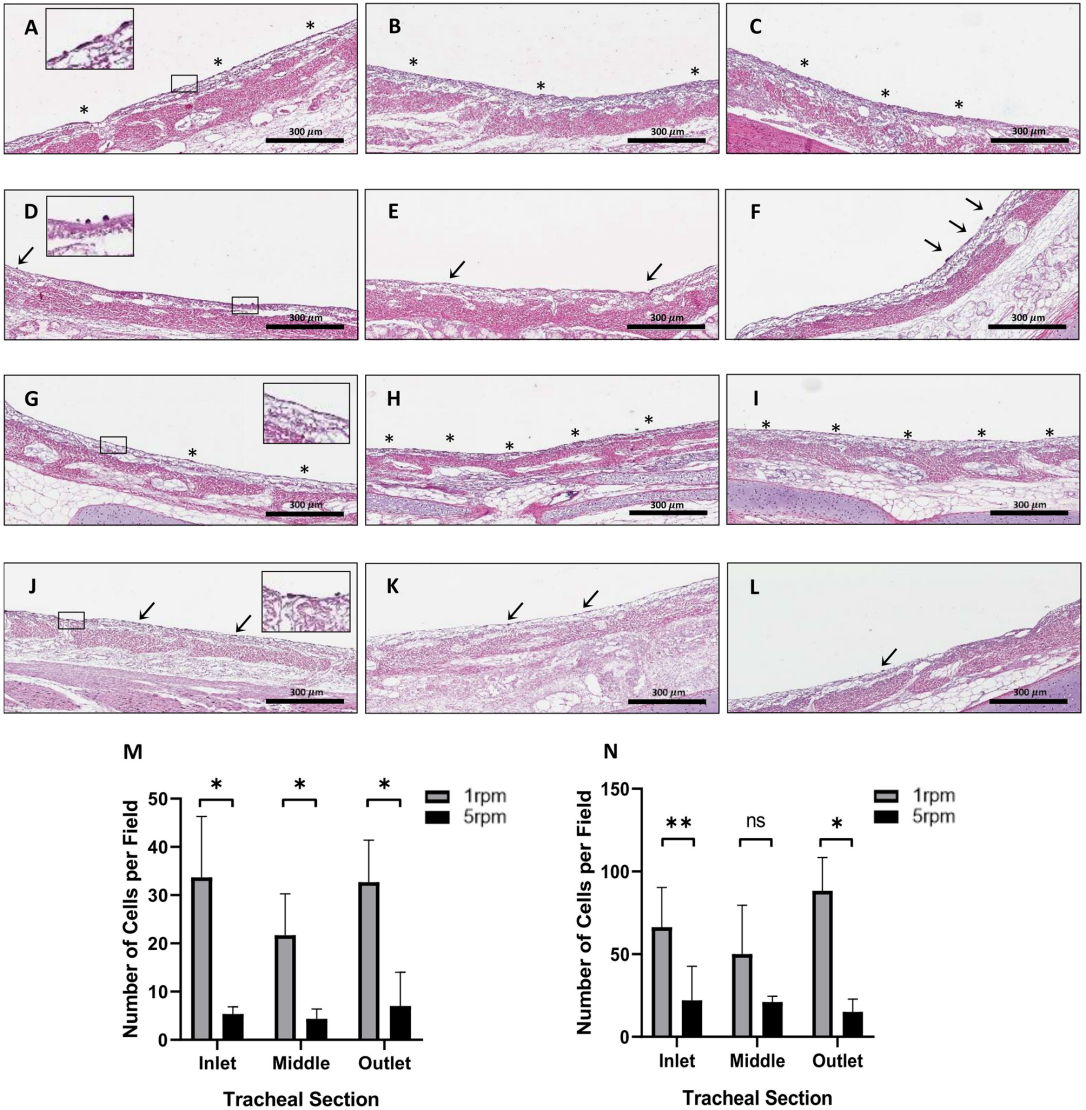
The H&E staining and cell quantification results comparing seeding at different rotational speeds in the single rotating inlet bioreactor. Inlet, middle, and outlet tracheal sections 2 h after seeding at (**A**–**C**) 1 rpm, and (**D**–**F**) 5 rpm; and 24 h after seeding at (**G**–**I**) 1 rpm, and (**J**–**L**) 5 rpm. Cell quantification results after (**M**) 2 h, and (**N**) 24 h in the bioreactor at 1 rpm (1.48e−04 Pa), and 5 rpm (4.11e-04 Pa). Data presented as means with standard deviations (n = 3 each group, ** p = 0.0024, * p < 0.02, ns = not significant with p > 0.05).

Quantification of cell number at the inlet, middle and outlet of tracheal grafts in the single rotating inlet bioreactor (Fig. S2C), suggested homogenous distribution of cells in the trachea at a rotational speed of 1 rpm. This is in contrast to cell distribution at a rotational speed of 5 rpm^9–11^. Encouraged by the ability of the simulation model to predict cell deposition patterns, both axially and circumferentially, at low rotation speeds, we carried out biological experiments (cell repopulation studies on de-epithelialized tracheal grafts) using simulation results as a guide. Given there was no significant influence of the flow rate on the wall shear stress and cell deposition in the trachea, we continued our experiments with our previously defined rate of 1.5 mL/min. For the rotational speed, we selected to test rotational speeds of 1 and 5 rpm. To evaluate the effect of rotational speed on initial cell attachment, tracheal grafts were seeded with BEAS-2B cells (1.0 × 10^6^ cells/cm^2^) and the number of cells were quantified at the inlet, middle and outlet of each graft at 2 and 24 h after cell seeding. Histological sections depicting inlet, middle and outlet of grafts seeded at a rotational speed of 1 rpm (Fig. 5A–C) show clear patches of BEAS-2B cell coverage on the luminal surface (asterisk) at 2 h after cell seeding. In grafts seeded with a rotational speed of 5 rpm, histological sections (Fig. 5D–F) show very sparse attachment (arrow). Quantification of cell number showed that there was a significantly higher number of cells attached in tracheal grafts in all regions of the trachea (Fig. 5M). At 24 h after cell seeding, histological images show a similar trend to that observed at 2 h with the grafts seeded at 1 rpm showing a much greater number of cells attached (Fig. 5G–I). In certain areas within each of the regions (asterisk) patches have converged and begun to form a monolayer. In grafts seeded with a rotational speed of 5 rpm, although there are more visible cells attached in all the regions (arrow), coverage still remains sparse (Fig. 5J–L). Quantification of cell number at 24 h, similarly, shows a higher number of cells in grafts seeded at 1 rpm which is significant at the inlet and outlet (Fig. 5N). We confirmed the viability of cells at both 2 and 24 h using live/dead staining (Fig. S5).

### Re-epithelialization of de-epithelialized long segment tracheal grafts

**Figure 6:**
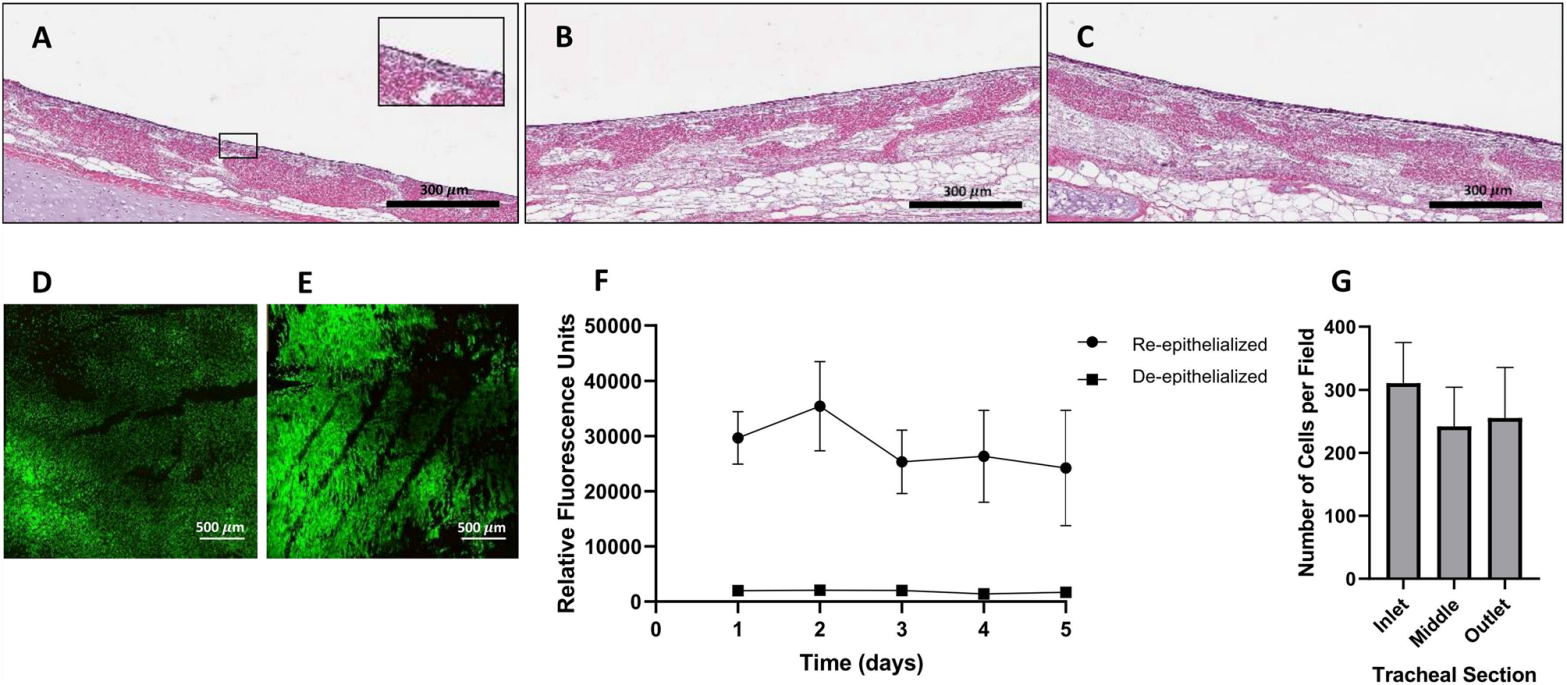
Long-term re-epithelialization of de-epithelialized tracheal grafts at 1 rpm in the single rotating inlet bioreactor. (**A**–**C**) H&E staining of the inlet (**A**), middle (**B**), and outlet (**C**) sections. (**D**,**E**) Live/Dead staining of the inlet (**D**) and outlet sections (**E**). Images depict calcein-AM for live (green) and ethidium homodimer-1 for dead (red) cells. (**F**) Metabolic cell activity over time compared to de-epithelialized tracheal grafts. Data presented as means with standard deviations (n = 3). (**G**) Cell quantification seeding results at 1 rpm (1.48e−04 Pa) after 5 days in the bioreactor. Data presented as means with standard deviations (n = 3, p > 0.05).

Using the acquired knowledge from our particle tracking model, and initial cell attachment studies, we selected a flowrate of 1.5 mL/min and tracheal rotational speed of 1 rpm for proof-of-concept long-term tracheal repopulation studies using BEAS-2B cells. Long-segment tracheal grafts (5 cm) were seeded with BEAS-2B cells (1.0 × 10^6^ cells/cm^2^) as per our established protocols. Grafts were evaluated over 5 days of culture in the enhanced bioreactor. Our bioreactor system allowed for continuous monitoring of parameters including temperature and flow. Moreover, the newly added sample ports allowed for daily evaluation of biochemical processes such as glucose consumption, lactate production and other metabolite concentrations (Figs. S6, S7). On day 5, grafts were harvested and evaluated for luminal cell coverage and cell viability. Histological evaluation of grafts shows complete coverage of the luminal surface in all regions (inlet, middle and outlet) (Fig. 6A–C). We evaluated the metabolic activity of cells (an indirect measure of cell viability and proliferation) over the 5 days (Fig. 6F) which showed that the cells were not only attached but metabolically active. In addition, we confirmed that the attached cells were alive via live/dead staining and showed no visible ethidium bromide red staining representing dead cells (Fig. 6D,E). Quantification of cells showed homogenous distribution of cells in the tracheal grafts with no significant differences between the regions (Fig. 6G). Taken together, our results show that our particle tracking model can be used at a guide for cell repopulation of tracheal grafts. The rotational speed selected based on the model resulted in enhanced re-epithelialization of grafts after long-term culture in the bioreactor.

## Discussion

A new, double-chamber, rotating bioreactor has been designed and fabricated for the de-epithelialization and re-epithelialization of tubular scaffold grafts. CFD has been used in this study to elucidate the differences in the tracheal graft wall shear stress profile caused by a design change of the bioreactor fluid delivery mechanism. The double static inlet design exhibited both more significant shear stress peaks near the inlet and the outlet and an unsteady shear stress profile compared to the single rotating inlet design. These findings illustrate how the flow field inside a bioreactor and its resulting mechanical environment of the tissue scaffolds is highly sensitive to the fluid delivery mechanisms of a bioreactor. The cause for the shear stress peaks near the inlet and outlet is likely from the edge effects at the bioreactor walls. Axial rotation of the trachea in the double inlet bioreactor design creates a rotating lumen wall while the interfacing cap walls remain static. This produces extra wall friction at the lumen wall near the static caps, corresponding to the shear stress peaks. A single rotating inlet design eliminates the static-rotating interfacing walls that share a common edge, by having rotation in all interfacing walls. Considering the sensitive nature of mammalian cell growth and stimulation to shear stress via mechano-transduction^15,24–33^, non-homogenous wall shear stress profiles is greatly undesirable for the culturing of a fully functional tissue-engineered tracheal scaffold. Hence, the rotating single inlet design exhibited a more consistent flow field environment for tracheal scaffolds than the double static inlet design.

Moreover, the wall shear stress distribution is much more sensitive to changes in the rotation rate than to changes in the flow rate, making the rotation rate the parameter of choice to control the mechanical environment inside the tracheal scaffold. The flow rate could be used to control the flow of nutrients and, if needed, to modify the flow streamlines and to control the cell deposition patterns during re-cellularization. Our results provide strong evidence that the rotation rate, an easily controllable experimental parameter, has a significant influence on the mechanical environment at the lumen wall and, thus, on the ability of the cells to deposit and subsequently attach to the tracheal scaffold. Although the bioreactor was originally designed with tracheal regeneration in mind, its potential applications can extend to culturing of tissue engineered tubular scaffolds including the esophagus, large blood vessels, gastrointestinal tract, and urinary tract.

The second part of this study used the Lagrangian based discrete phase model to predict the spatial cell deposition patterns on the tubular scaffold lumen (tracheal graft). Although the gold standard for tracheal recellularization is bi-directional peristaltic cell seeding, considerations of computational feasibility led us to select a steady-state formulation to model unidirectional flow. Our rationale was that this simulates the first pass forward of a bi-directional seeding protocol, and thus provides a baseline, a lower bound on the cell deposition predicted by the simulations. The results of the particle tracking showed that there was negligible effect of flow rate on cell deposition. This contrasted with rotation which resulted in significant changes in the wall shear stress distribution and the flow structure, as evidenced by the flow streamlines. Induction of rotation caused an increasingly circumferentially uniform cell deposition pattern along the tubular scaffold. This shows that centrifugal forces from the tracheal rotation played a significant role on the trajectory of particles, causing the particles to move outwards from the inlet onto the lumen surface. Overall, the particle accretion results were observed to be very sensitive to the bioreactor inlet and outlet designs, as was to be expected from their dependence on the flow field, and to be more sensitive to the rotation rate than to the flow rate, a perhaps unexpected result that reveals a robust mechanism to control the flow field and cell deposition patterns inside the scaffold.

The third part of the study focused the experimental validation of the cell tracking model. The effect of gravity that caused a circumferential bias towards the bottom of the trachea predicted by the cell tracking simulation model is well captured in the 0, 1 and 5 rpm validation results. The relatively large variability exhibited for the 0 rpm and 1 rpm cases, may be due to clumping effects from cell-to-cell interactions, which would increase the effective size of the particles and reduce the number of independent particle trajectories. This will result in a significant proportion of the cell deposition fraction to be concentrated in a localized area. Cell clumping is an important factor that was disregarded in the simulations and has been frequently observed in our experimental work in tracheal graft repopulation. Induction of rotation causes the longitudinal trends of the simulation to deviate from the experimental results. At 1 rpm, the experimental longitudinal trend exhibits a relatively steady and level trend from the inlet to the outlet while the simulation predicted an unchanging declining trend. Beginning at 5 rpm, the longitudinal trend of the simulation shows a sharp contrast to the experimental results where an increasing cell deposition trend is seen as opposed to a steadily declining trend. The differences between the axial (i.e. length-wise) deposition patterns in experimental and simulation results at moderate and large rotational speeds, combined with the ability of the simulation to predict the axial deposition patterns at low rotational speeds and the circumferential deposition patterns at both low and moderate rotational speeds, suggests that the simulation model does not include all the relevant physical phenomena. This suggests that at higher wall shear stresses induced by higher rotational velocities, the cell-scaffold interaction deviates away from the attachment-upon-contact assumption. This trend is indicative of biological cell-scaffold interactions that are shear stress sensitive which were not accounted for in the simulations^25^.

Furthermore, it has been documented that the use of harsh detergents (e.g., SDS), such as those used in our decellularization protocol (described by Aoki et al*.*^11^), can remove extracellular matrix proteins that play a vital role in cell attachment for tracheal epithelial cells^6,36–38^. In this case, the simulations would predict a larger cell deposition rate, while in reality, the bio-chemical environment would not have been favorable for the cells to attach onto the luminal surface of tracheal graft. Overall, the results of the validation experiments illustrate the limitations of using a simple particle tracking CFD model without consideration of complex cell-to-cell and cell-to-scaffold interactions^25^, and the sensitivity of the cell to its biochemical and hydrodynamic environment^15,34,35^. Despite these limitations, the model was shown to successfully predict general trends in a low-shear-stress-hydrodynamic environment.

The simulation results shown in this work are steady state results of unidirectional perfusion cell seeding flow. A more accurate depiction of the experimental cell seeding conditions would require a transient simulation that considers the pulsatility of the flow caused by the peristaltic pumps and the periodic reversing of the flow to see the real time effects of these factors that play in the cell deposition and attachment processes during the bi-directional, perfusion cell seeding protocol that is used in our tracheal re-epithelialization protocols. However, the computational cost of such simulations is prohibitive. For instance, using the same computational mesh and a temporal discretization with a time step of $1\times {10}^{-2}$ seconds, simulating bidirectional flow for 8 min, would require approximately 6,400 core hours of compute time. Another aspect of the simulations that could be improved is the handling of wall-particle interactions. In this work, the ‘trapped’ condition between the discrete particle and the trachea lumen wall was used for the simulation. This implies that an epithelial cell in suspension will adhere onto the trachea lumen surface upon contact regardless of the surface hydrophobicity, potential charge, chemical composition, wall stiffness, angle of incidence or particle impact velocity, all of which may play a role in the adhesion of cells on a scaffold surface. This assumption is unrealistic in practice, particularly when the particles in questions are living cells impinging on a decellularized tissue scaffold. Hence, more realistic cell-scaffold interactions must be used in the future to more accurately model the cell deposition and attachment process, perhaps including cell–cell interactions that synergistically favor both deposition (through cell clumping) and attachment (through biochemical signaling). Of interest in this context are interaction models that are a function of both instantaneous and time-dependent quantities.

A uniform hydrodynamic environment for tracheal grafts has been made possible with a CFD optimized bioreactor design. A CFD model for particle tracking has modeled the spatial cell deposition distribution of the perfusion cell seeding protocol. Experimental validation of cell deposition shows good agreement at low shear stress environments, which becomes progressively more error ridden as the rotation speed/shear stress increases, suggesting the need for better models that include the biomechanics of cell attachment. The hydrodynamic environment of tubular scaffolds inside a double chamber, rotating bioreactor is highly sensitive to the fluid delivery system of the bioreactor. Even at low rotational velocities, centrifugal forces cause the cell trajectory to move towards the scaffold wall thereby causing cell attachment. Based on our results, the fluid delivery system of the bioreactor that promotes uniformity of the flow fields at the lumen wall, an average wall shear stress environment of below 0.0002 Pa with a rotational velocity of about 1 rpm to allow a circumferentially uniform cell deposition on a tubular graft construct is recommended for perfusion cell seeding of cells within a bioreactor.

Our re-epithelialization studies were re-designed based on guidance from the CFD simulations and particle tracking model. Cell repopulation using conditions resulting in low wall shear stress enabled enhanced re-epithelialization of long segment tracheal grafts. This has significant implications for the tracheal regeneration field as one of the critical limitations is inadequate repopulation of tracheal scaffolds. While our work focuses on tracheal regeneration, lessons learned in this study, can be applied to culturing of any tissue engineered tubular scaffold.

## Materials and methods

Experiments were performed in accordance with the guidelines and regulations of the of the Toronto General Hospital Research Institute. All animal studies were fully approved by the Toronto General Hospital Research Institute Animal Care Committee and ethical review board under protocol AUP6130. Humane care was provided to all animals in conformity with the “Principles of Laboratory Animal Care” defined by the National Society for Medical Research and the “Guide for the Care of Laboratory Animals” as issued by the National Institutes of Health.

### Bioreactor design and manufacture

The trachea bioreactor designed in this work is an electromechanical device consisting of the culture chamber and the electronic control unit (not shown). It is schematically represented in Fig. 1 showing isometric (Fig. 1A), side (Fig. 1B), cross-sectional (Fig. 1C) and exploded (Fig. 1D) views. The culture chamber is fabricated using a translucent autoclavable polymer called polyphenylsulfone (Poly-Tech Industrial Inc., USA) and stainless-steel 316 assembled together with off-the-shelf components including a swivel joint adapter, timing belt pulleys, timing belt, plain bearing, O-rings, shaft collars, polypropylene zip-ties and barbed adapters. The disassembled parts, excluding the electronics, can be sterilized inside an autoclave. The trachea is securely tightened between two rotating anchors and the two ends of the trachea fit over each anchor with grooved tips to prevent slippage. The position of the long anchor with respect to the body can be adjusted to accommodate for tracheas of various lengths from 3 to 10 cm. Once the trachea is secured onto the two opposing anchor pieces, the tracheal graft wall isolates the interior lumen chamber from the exterior culture chamber. A modular inlet in the bioreactor culture chamber allows additional control over the flow field of the inner chamber. Figure 1C shows a cross section of the single rotating inlet and the double static inlet that the bioreactor platform is compatible with. Note that these two inlet designs differ not only on the number and position of the entry ports, but also on the relative motion between the (left) long anchor and the trachea. In the single-inlet design, a swivel joint adapter is used which allows the inlet port, anchor and the trachea to rotate together with respect to the external tubing that supplies the cell media to the bioreactor. In contrast, in the double-inlet design, the entry ports and long anchor are fixed, and only the trachea rotates with respect to them. The design approach for the outlet is common to both single-inlet and double-inlet cases, however, consisting of a stationary short anchor around which the trachea rotates, with the anchor connecting to the external tubing through one (single-outlet) or two (double-outlet) exit ports through a barbed adapter without a swivel joint.

The electronic control unit includes the actuators, sensors, microcontrollers and a laptop (Fig. S1). The DC motor allows tracheal rotation along its longitudinal axis from 0 to 30 rpm. The rotation allows circulation and mixing of micronutrients, provides control of the lumen wall shear stress, positional control during perfusion cell seeding and a periodic exposure to both air and liquid when an air–liquid–interface is achieved in the inner chamber. Microcontrollers control the rotational velocity of the trachea and the flow rate of the custom peristaltic pumps. An inline flowmeter (Sensirion, Staefa, Switzerland), and a stainless-steel housed thermistor (QTI Solutions, Boise, United States) from the bioreactor are connected directly to a laptop via USB for data acquisition and real time data display of its flow and temperature data. A 3-way Luer stop-cock valve located at the lumen chamber outlet allows for sample fluid collection via a syringe.

### CFD approach and mesh design

**Figure 7:**
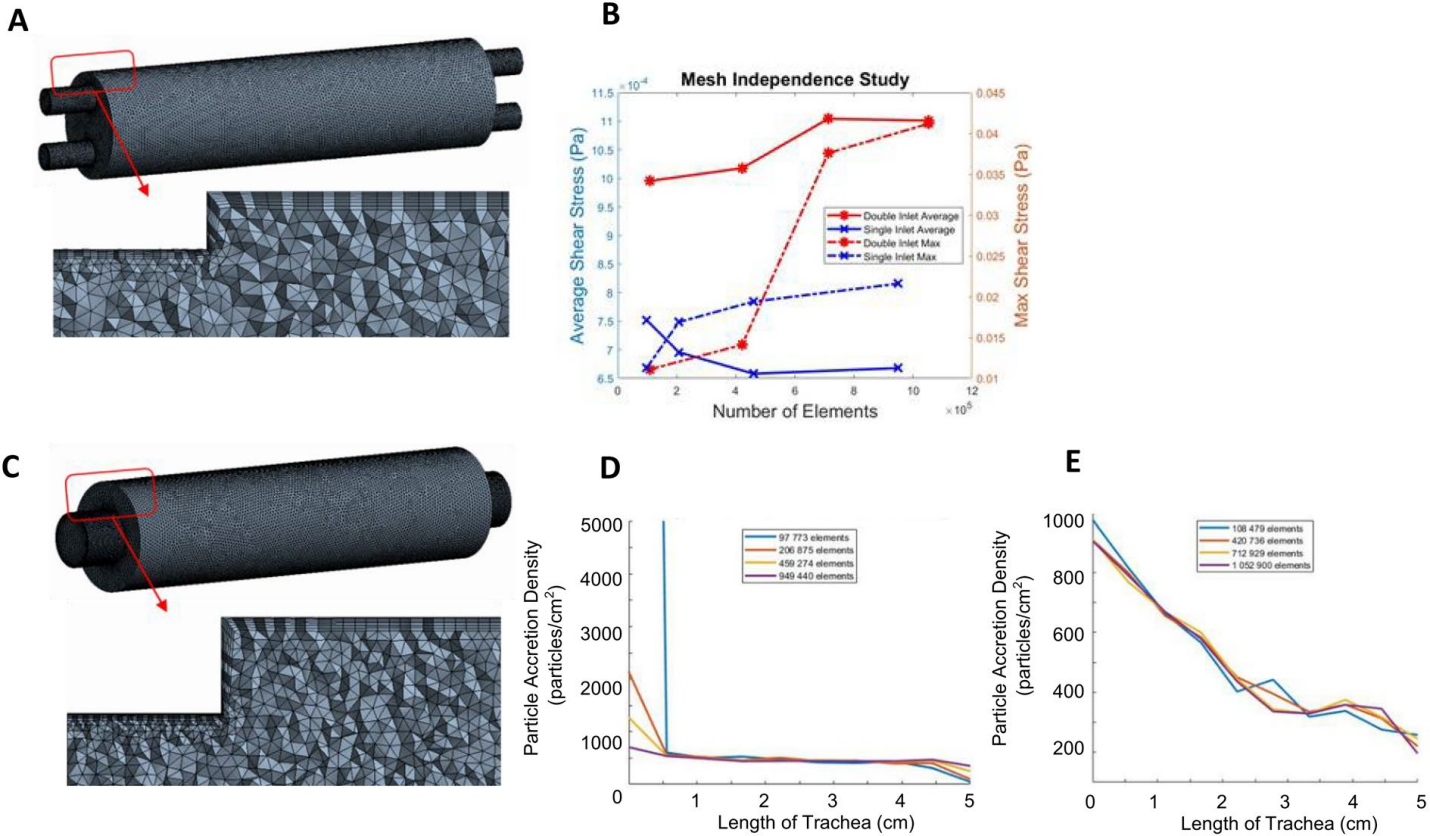
Finalized mesh of the bioreactor inner chamber. (**A**) Double static inlet bioreactor design meshed geometry and its magnified cross sectional view of the mesh highlights the inflation layers near the boundaries; (**B**) Mesh independence study plot of the average lumen wall shear stress at 15 rpm of rotational velocity for the double inlet (blue) and single inlet design (red); (**C**) Single rotating inlet bioreactor design meshed geometry and its magnified cross-sectional view; Mesh independence plot showing spatial uniformity of particle accretion at the finalized mesh for the (**D**) single and (**E**) double inlet designs. (**A**) and (**C**) were generated using ANSYS Workbench v19.1 (ANSYS Inc., Cannonsburgh, PA, USA; https://www.ansys.com). (**B**), (**D**) and (**E**) were generated using MATLAB 2018b (Mathworks Inc., Natick, Massachusetts, USA; https://www.mathworks.com/).

The dimensions of the bioreactor interior chamber were used to generate a 3D geometry of the fluid domain using Solidworks v2016 (Dassault Systemes, Vélizy-Villacoublay, France). The total fluid volume of the entire bioreactor interior chamber geometry used for the simulation is 6.52 mL. Volumetric fluid meshing was performed using ANSYS Workbench v19.1 (ANSYS Inc., Cannonsburgh, PA, USA). Since velocity gradients will have the highest magnitude at the inlet, outlet and at the lumen walls, these regions were assigned 7 smooth inflation layers at the walls and a more refined mesh (Fig. 7A,C). A mesh independence study was performed to ensure that the numerical solutions were independent of the mesh size. The average and maximum shear stress across the entire lumen (Fig. 7B), and the average particle accretion rate along the length of the lumen (Fig. 7D,E) were used as the mesh independence criteria. For the double static inlet geometry, a mesh with 1,052,900 elements resulted in less than 2% change in the average shear stress and particle accretion rate, and a 5% change in the maximum shear stress with respect to a mesh with 712,929 elements, so the former was selected as the final mesh for the double inlet simulations. For the single rotating inlet geometry, we selected a mesh with 949,440 elements, which resulted in less than 2% change in average shear stress and particle accretion rates, and a 10% change in the maximum shear stress with respect to a mesh with less than half of the elements (459,274 elements).

CFD simulations were conducted using FLUENT v. 19.1 (ANSYS Inc., Canonsburg, PA, USA). The culture medium, which normally fills the entire inner bioreactor chamber, was modelled as a Newtonian fluid, set to a temperature of 37^o^ C, viscosity µ of 10^–3^ Pa*s, density ρ of 10^3^ kg/m^3^, with hydrodynamic behaviour described by the Navier Stokes and continuity equations:

1 $$\nabla \cdot \overrightarrow{u}=0$$

2 $$\rho \overrightarrow{u}\cdot \nabla \overrightarrow{u}=-\nabla p+\mu {\nabla }^{2}\overrightarrow{u}$$

where $\nabla$ is the del operator, $\overrightarrow{u}$ is the fluid velocity vector, $\rho$ is the density of the fluid within the fluid domain, p is the pressure and $\mu$ is the dynamic viscosity of the fluid.

The re-epithelialization process that occurs within the inner chamber of the trachea bioreactor was simulated using a Lagrangian formulation for cell tracking, i.e., using the discrete phase model (DPM). The discrete phase in the simulation, representing cells, were modelled as inert spherical particles whose trajectory is dominated by gravity and by the drag forces exerted by the fluid on the particles. Given the small density difference between the particles and the flow, and the small volumetric fraction represented by the particles, the forces that the particles exert on the fluid were not included in the simulations (one-way coupling). A sensitivity study to this assumption was conducted (not shown), with two-way coupling resulting in a three-fold increase in computational time and less than 1% change in the observed endpoints. The particle trajectory was calculated by integrating the forces acting upon an individual spherical cell in a media suspension within a bioreactor, as shown by:

3 $$\frac{{du}_{p}}{dt}={F}_{D}\left(u-{u}_{p}\right)+\frac{{g}_{x}\left({\rho }_{p}-\rho \right)}{{\rho }_{p}}$$

4 $${F}_{D}=\frac{18\mu }{{\rho }_{p}{d}_{p}^{2}}\frac{{C}_{D}Re}{24}$$

where F_D_ is the spherical drag force acting on the particle, the magnitude of which is proportional to the difference in velocity between the fluid (u) and the particle (u_p_), g_x_ is the gravitational constant, ${\rho }_{p}$ the density of the particle, $\rho$ the density of the media, µ the dynamic viscosity of the fluid, C_D_ the drag coefficient, Re the Reynolds number of the flow and d_p_ the diameter of the cell particle.

### Boundary conditions and simulation parameters

The Navier Stokes and continuity equations for the continuous phase shown in Eqs. (1) and (2) were solved for a steady state, incompressible and laminar flow case, consistent with the flow rates typically used for recellularization experiments (1.5 mL/min) which yield a Reynold’s number of approximately 1 based on a tracheal diameter of 12.7 mm. A fully developed, non-pulsatile flow was set at the bioreactor inlet, with a 0-pressure outlet and a no-slip condition at the remaining boundary walls, which were assigned a tangential velocity consistent with rotation speeds of 0, 1, 5 and 15 rpm.

For the DPM, spherical particles with a particle diameter of 10 µm and particle density ${\rho }_{p}$= 1050 kg/m^3^^21^ representing epithelial cells suspended in media were randomly distributed across the inlet channel. To account for the potential effect of the initial particle positions on the simulation results, we repeated each simulation three times (n = 3 ) with different sets of initial particle positions. Simulation results were essentially insensitive to the initial particle positions, with observed changes below 1%. Regardless, error bars are included in simulation results where appropriate (e.g., Fig. 4). A sensitivity study was conducted to determine the number of particles to include in the simulation, monitoring quantitative changes in the percentage of deposited particles and qualitative changes in particle accretion maps. Simulations with 110,000, 11,000 and 1100 particles were conducted, with results (not shown) indicating that 11,000 particles were sufficient to obtain results that are independent of the number of particles. Hence, results shown in this manuscript were obtained with 11,000 particles, each particle models 100 epithelial cells to match the cell concentration of 24 million cells/mL used during re-epithelialization experiments in our research group. The trajectory of the particles was integrated using a step size of 0.001 m, with the maximum number of possible particle steps set up at 500,000 steps, thus setting the maximum length of any particle trajectory to 500 m; this was deemed sufficient to ensure that each particle was tracked until it either deposited on the lumen wall or exited the calculation domain. To model the cell attachment of the individual cells onto the tracheal graft, a *trapped* boundary condition at the lumen wall was used. The *trapped* wall-particle condition assumes that any particle that physically collides with the trachea lumen wall attaches or accretes onto the lumen surface. Hence, upon impact, the trajectory calculation of the particle is terminated. Any cell particle that does not make contact with the tracheal graft lumen surface and travels as far as the outlet is considered to have *escaped* the domain. The remaining cell trajectories which remain inside the fluid domain without escaping or being trapped after the maximum set particle steps are labelled as *incomplete* trajectories.

### Experimental validation

For validation of our model, we focused on trends, rather than traditional quantitative measures of accuracy. Due to complexities associated with in vitro experiments with live cells, particularly with epithelial cells, traditional quantitative measures which would be a poor indicator of model utility given the knowledge gap regarding cell response to the uncertain local mechano-biological environments on which they deposit.

Unidirectional perfusion cell seeding as simulated using our CFD model was performed for validation. Experiments were performed at 0 rpm, 1 rpm, 5 rpm and 15 rpm, with three replications of each experiment (n = 3). The validation experiments began with the surgical removal^10^ of a 5 cm tracheal graft segment from a Yorkshire pig which was then de-epithelialized following Aoki et al*.*’s protocol^11^. This trachea was later attached onto the bioreactor which was placed inside an incubator at 37^o^ C. The flow circuit to the inner chamber began with one full 60-mL syringe connected to a syringe pump (AL-4000, *World Precision Instruments*), the media exited onto a waste bottle to form an open loop flow circuit. The waste flask at the end of the circuit collected all the ejected fluid from the bioreactor outlet. Centrifugation and cell counts were performed to determine the number of cells that escaped the bioreactor without adhering onto the tubular scaffold.

To allow for steady state conditions of the flow field inside the bioreactor chamber, the inner chamber was set to rotate 5 min (in the 1 rpm and 15 rpm cases, where rotation was applicable) before starting the media flow. Then, continuous one-way flow at 1.5 mL/min was started and sustained for 5 min using a syringe pump, to establish a steady-state flow field inside the tracheal scaffold. Subsequently, approximately 20 million BEAS-2B cells, prepared in a 1 mL cell suspension, were injected through a 3-way stop-cock located 12 cm from the trachea inlet using a 6-mL syringe. One-way flow at 1.5 mL/min then followed for a period of 23 min, which was determined to be sufficient to allow for suspended cells to deposit onto the wall or exit the trachea through the outlet. After these 23 min of flow post-injection, both flow and rotation were stopped for 3 h and 37 min to allow for the cells to attach onto the tracheal grafts. At this point, the trachea scaffold was removed from the bioreactor, fixed using a paraformaldehyde (PFA) solution and bio-punched. A 6 mm diameter bio-punch was used to get 25 equally spaced circular punches across the entire tracheal lumen surface (Supplementary Fig. S6). Peeled segments of submucosa were stained with HOECHST 33258, washed and imaged using fluorescent, confocal microscopy (A1R, *Nikon*, Tokyo, Japan) at 20 × magnification.

### Animals and tracheal harvest

Tracheal samples were collected as previously described^10,11^. Briefly, Yorkshire pigs (3 months of age and ranging in weight from 28 to 35 kg) were anesthetized using an intramuscular injection of a mixture of ketamine (25 mg/kg), atropine (0.04 mg/kg), and midazolam (0.15 mg/kg). Prior to harvest of the grafts, pigs were maintained at 5% isoflurane. The animals were sacrificed using an overdose of isoflurane and propofol and tracheal grafts were collected under sterile conditions^10^ and stored in Hank’s balanced salt solution (HBSS) containing 2% bovine serum albumin (BSA) and an antibiotic–antimycotic cocktail composed of fluconazole (4 µg/mL), colistimethate (5 µg/mL), imipenem/cilastatin (25 µg/mL), ceftazidime (154 µg/mL), penicillin (200 U/mL), streptomycin (200 µg/mL), amphotericin B (0.5 µg/mL) and gentamicin (50 µg/mL). Grafts were stored at 4^0^C until subsequent use.

### Tracheal graft de-epithelialization

De-epithelialization was performed as previously described^11^. Briefly, a 1% SDS (w/v) in deionized water (diH_2_0) was run through the inner luminal chamber of the rotating bioreactor for 3 h to remove the tracheal epithelial cells. After the 3 h SDS treatment, the inner luminal chamber was perfused with diH_2_O (40 min), 1% Triton X-100 in diH_2_O (30 min), and phosphate buffered saline (PBS; 30 min). To maintain the cartilage, the outer chamber was filled with growth media, Dulbecco’s modified Eagle’s medium (DMEM, Gibco, USA), supplemented with 10% (v/v) fetal bovine serum (FBS; Gibco, USA) and 1% (v/v) penicillin–streptomycin (Gibco, USA).

### Cell culture

BEAS-2B cells (CRL-9609, ATCC, USA) were cultured as per previous studies in our lab^11^. Briefly, cells were cultured in DMEM media supplemented with 10% (v/v) FBS, and 1% (v/v) penicillin/streptomycin. Experiments were performed using BEAS-2B cells between passage 30 and passage 35, with passage 1 defined as the thawed cells from the supplier. Cells were passaged using Trypsin–EDTA (0.25%) to lift and remove cells from culture flasks. Cell cultures were maintained in an incubator in the presence of humidified 95% air and 5% CO_2_. Two million cells were seeded in 75 cm^2^ culture flasks and passaged at 90% confluence.

### Tracheal graft cell seeding and recellularization

Cell seeding on de-epithelialized tracheal grafts was performed as previously described by our group^11^. Briefly, de-epithelialized tracheal grafts were decontaminated in HBSS containing 2% (w/v) BSA and a broad-spectrum antibiotic solution for 48 h. Grafts were then reattached to the bioreactor and a 1 mL suspension containing 1 × 10^6^ cells BEAS-2Bs per cm^2^ of graft BEAS-2Bs was injected into the tracheal lumen. Recellularization was done by bidirectional perfusion cell seeding which involves the use of the peristaltic flow pump to inject the cell suspension into the inner chamber through the stop-cocks connected to silicone tubing for this chamber. Peristaltic bidirectional flow was utilized for the first 2 h allowing for cell adherence following which unidirectional flow was utilized towards the reservoir containing media. Media in the inner circuit (tracheal lumen) was changed every 24 h and half of the media in the outer circuit was changed every 48 h.

### Sample collection

Media samples were taken with 1-mL syringes from both chambers daily to determine changes in pH, glucose, and lactate. RAPIDPoint 500 Blood Gas Systems (Siemens Healthcare Limited, Canada) was used to measure these parameters in media samples. This analyzer can measure pH, partial pressure of carbon dioxide and oxygen, and concentrations of many ions such as sodium, potassium, chloride, bicarbonate, and metabolites, namely calcium, magnesium, glucose, lactate.

### Cell metabolic activity assay

Before changing the media of the intraluminal chamber of the bioreactor, cell metabolic activity was evaluated using a resazurin-based redox metabolic assay (PrestoBlue Cell Viability Reagent, Invitrogen by Thermo Fisher Scientific, Oregon, USA) as per our previously established techniques^11^ and manufacturer’s instructions. The resazurin reduction perfusion assay was performed in seeded tracheas daily. Briefly, PrestoBlue reagent was diluted at 1:20 (v/v) in the medium used for culture until a 20-mL resazurin-medium mixture was obtained, with 2 mL of the resazurin-medium mixture saved as a non-metabolized control before the remaining mixture was added to the intraluminal chamber of tracheal bioreactor. The seeded trachea was then perfused with resazurin-medium mixture at a rate of 1.5 mL/min for 1 h at 37 °C. The fluorescence of resazurin-medium mixture was measured and calculated using a Cytation5 Cell Imaging Multi-Mode Reader (BioTek Instruments, Inc., Winooski VT, USA) at 560 nm (ex)/590 nm xs(em).

### Histology, nuclear and live/dead staining

Histological evaluation was performed as per our previous studies^6,8,10,11^ with some modifications in sample preparation. Following 5 days of bioreactor culture, grafts (n = 3 for each group) were divided into three different blocks representing the inlet, the middle, and the outlet (approximately 0.8 cm sections) for histological evaluation. From the remaining graft, two segments (inlet and outlet) were prepared for live/dead staining. Tracheal samples were fixed in 10% neutral buffered formalin solution (for 48 h at room temperature), washed in distilled water, dehydrated in graded alcohol, embedded in paraffin, and sectioned at 5 µm thickness. Sections were then stained with hematoxylin and eosin (H&E) using standard techniques in the lab^10,11^. Briefly, deparaffinization of tissue sections was performed by three 3 min xylene changes with subsequent rehydration in an ethanol series. Sections were then exposed to hematoxylin for 1 min, rinsed with water, exposed to eosin, and rinsed with water a second time. Finally, slides were dehydrated with an ethanol series, exposed to xylene, and sealed with a coverslip.

For HOECHST nuclear staining, sections (following deparaffinization) were stained with HOECHST at a dilution of 1:300 in H_2_O, incubated for 5 min at room temperature, mounted with fluorescent mounting media (DAKO) and placed on coverslip. A Nikon A1R scanning microscope was used for confocal image capture.

Cell viability was evaluated using a LIVE/DEAD Viability/Cytotoxicity Kit (Invitrogen by Thermo Fisher Scientific, Oregon, USA). Inlet and outlet segments of the recellularized grafts were collected after the last metabolic activity measurement and placed in warm DMEM. Three samples were taken from each inlet/outlet segment using a 6 mm bio-punch. The mucosa was carefully detached from the cartilage and stained for 10 min at room temperature as previously described^11^ and per manufacturer instructions. A Nikon A1R scanning microscope was used for confocal image capture.

## Supplementary Information

Supplementary Figures.

## Data Availability

The data that support the findings of this study are available from the corresponding authors upon request.
