# Supplementary material for: Computational fluid dynamics for enhanced tracheal bioreactor design and long-segment graft recellularization
Source: Sci Rep. 2021 Jan 13;11:1187. doi: 10.1038/s41598-020-80841-w (PMC7807076; doi:10.1038/s41598-020-80841-w)
Supplement: Supplementary file 1 — Supplementary Figures. [file 41598_2020_80841_MOESM1_ESM.docx]

**COMPUTATIONAL FLUID DYNAMICS FOR ENHANCED TRACHEAL BIOREACTOR DESIGN AND LONG-SEGMENT GRAFT RECELLULARIZATION**

**^1*^Hankyu Lee, ^2,3*^Alba E. Marin-Araujo, ^3,4^Fabio G. Aoki, ^3,5^Siba Haykal, ^2,3^Thomas K. Waddell, ^1,2^Cristina H. Amon, ^1**^David A. Romero and ^1,3**^Golnaz Karoubi**

^1^Department of Mechanical and Industrial Engineering, University of Toronto, 5 King’s College Road, Toronto, Ontario M5S 3G8, Canada

^2^Institute of Biomaterials and Biomedical Engineering, University of Toronto, 164 College Street, Toronto, Ontario M5S 3G9, Canada

^3^Latner Research Laboratories, Division of Thoracic Surgery, University Health Network, 101 College Street, Toronto, Ontario M5G 1L7, Canada

^4^Institute of Science and Technology, Federal University of Sao Paulo, R. Talim, 330, Sao Jose dos Campos, SP 12231-280, Brazil

^5^Division of Plastic & Reconstructive Surgery, University Health Network, University of Toronto, 200 Elizabeth Street, Toronto, Ontario M5G2C4, Canada

* Authors contributed equally to the work

** Co-senior authors

§ To whom correspondence should be addressed ([d.romero@utoronto.ca](mailto:d.romero@utoronto.ca) & golnaz.karoubi@uhnresearch.ca)

**SUPPLEMENTARY INFORMATION**

**SUPPLEMENTARY FIGURES**


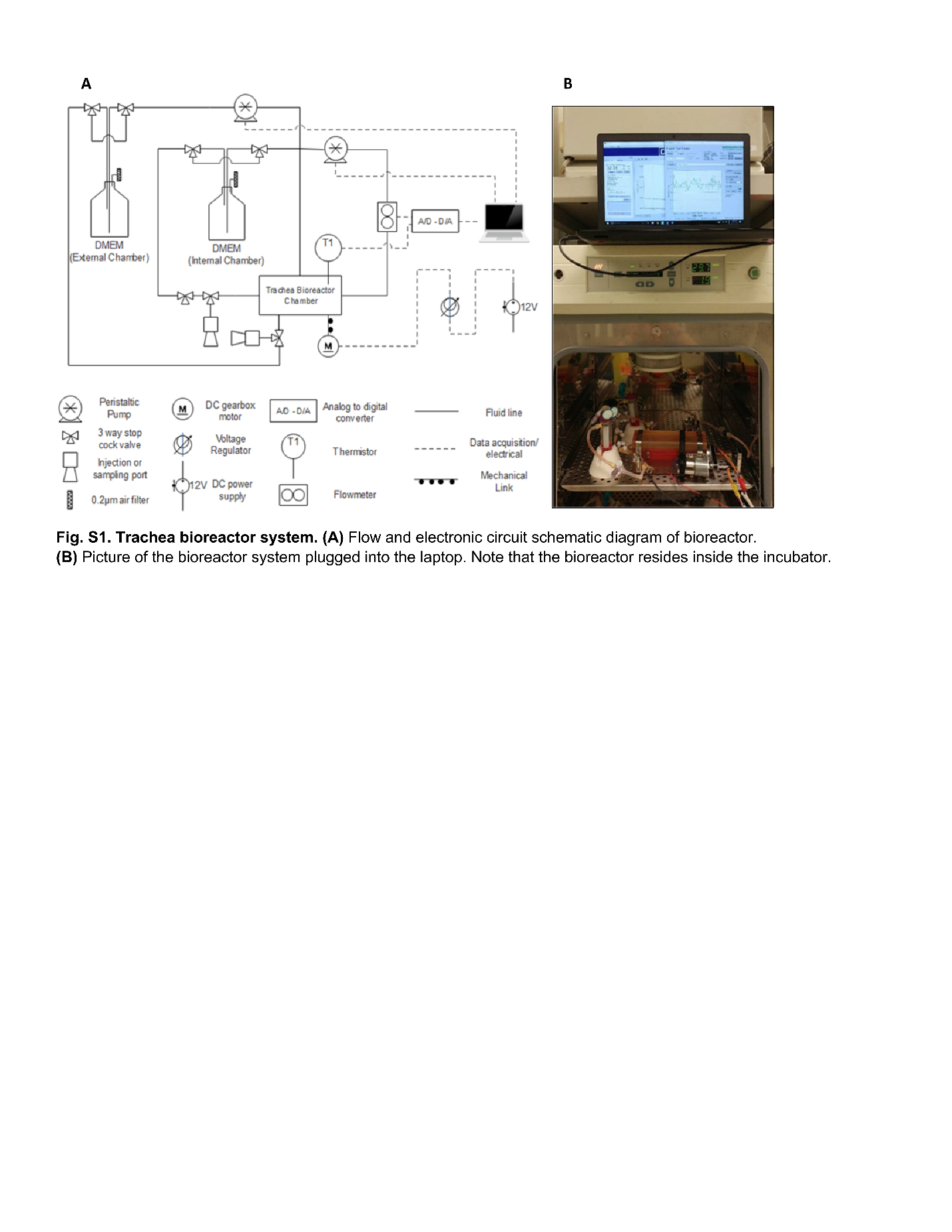


**Fig. S1. Trachea bioreactor system. (A)** Flow and electronic circuit schematic diagram of the bioreactor. **(B)** Picture of the bioreactor system plugged into the laptop. Note that the bioreactor resides inside the incubator.


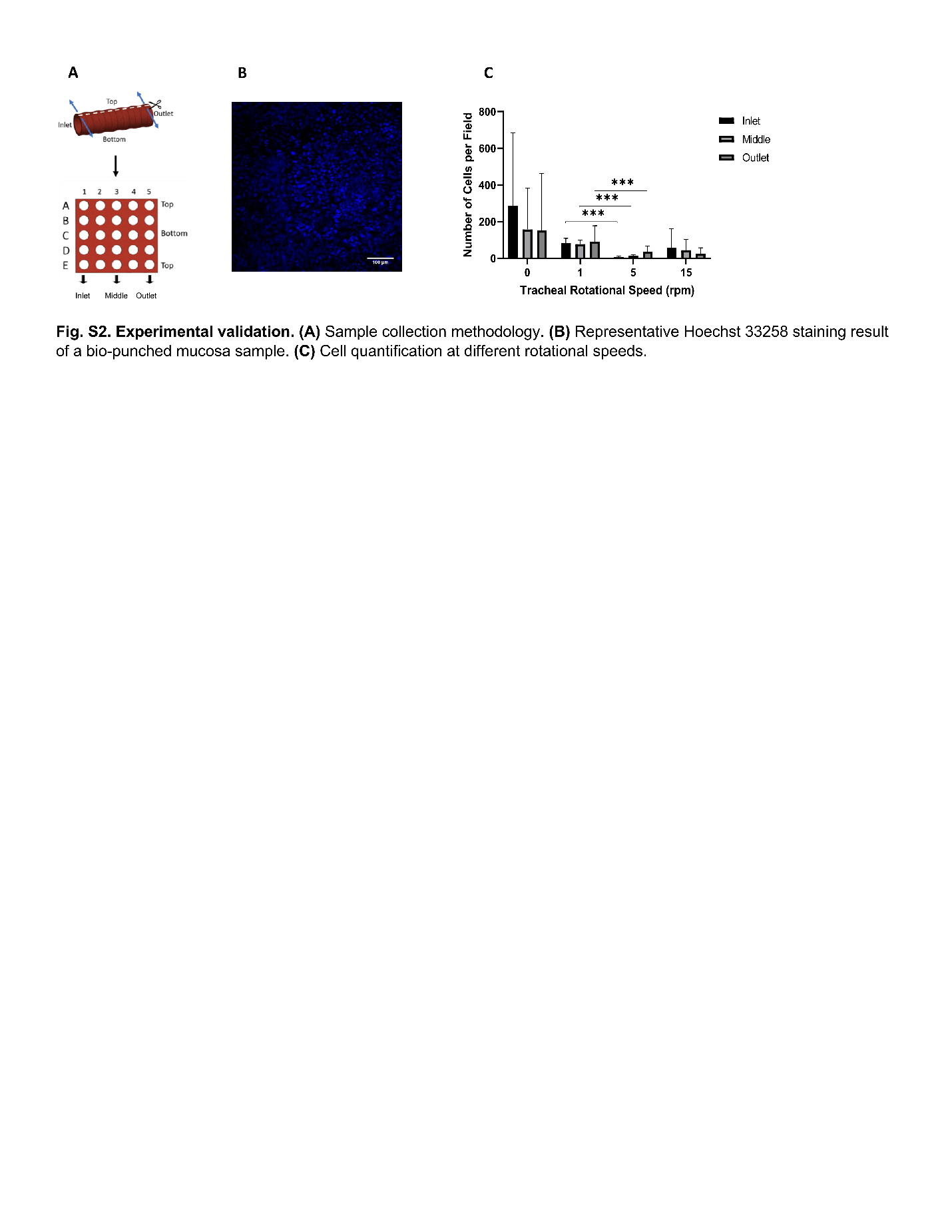


**Fig. S2. Experimental validation. (A)** Sample collection methodology.
**(B)** Representative Hoechst 332558 staining result of a bio-punched mucosa sample. **(C)** Cell quantification at different rotational speeds.


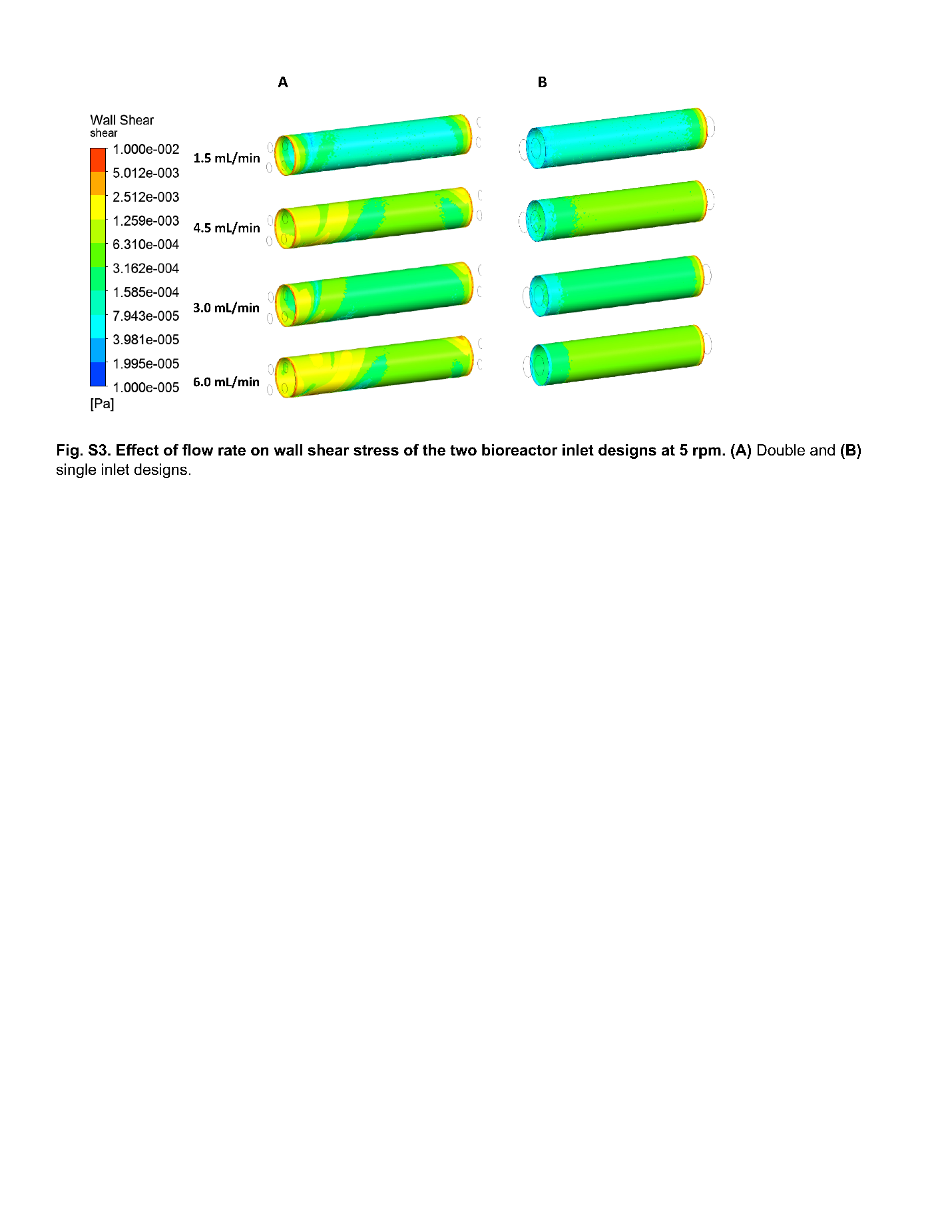


**Fig. S3. Effect of flow rate on wall shear stress of the two bioreactor inlet designs at 5 rpm. (A)** Double and **(B)** single inlet designs. Figure was generated using ANSYS Workbench v19.1 (ANSYS Inc., Canonsburgh, PA, USA; https://www.ansys.com).


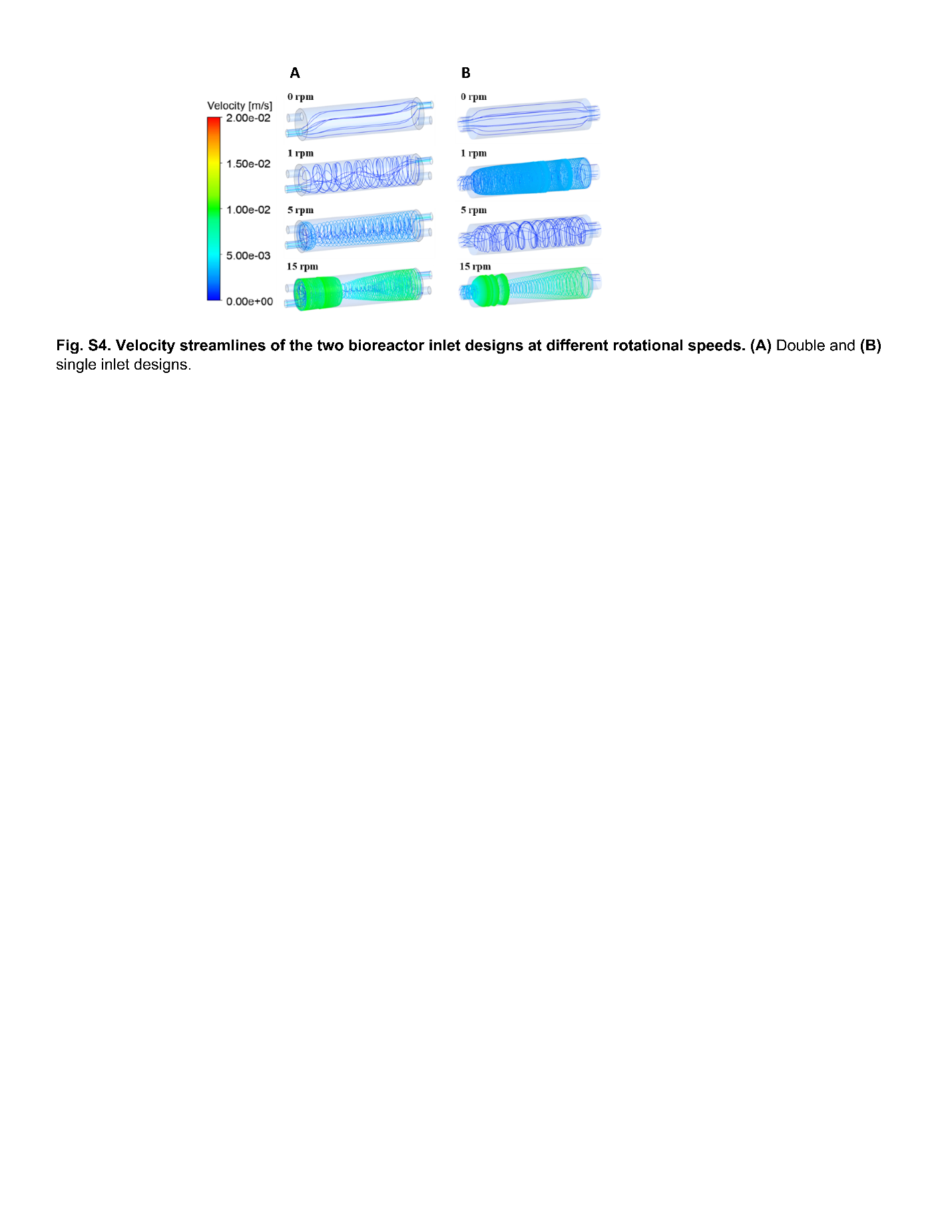


**Fig. S4. Velocity streamlines of the two bioreactor inlet designs at different rotational speeds. (A)** Double and **(B)** single inlet designs. Figure was generated using ANSYS Workbench v19.1 (ANSYS Inc., Canonsburgh, PA, USA; https://www.ansys.com).


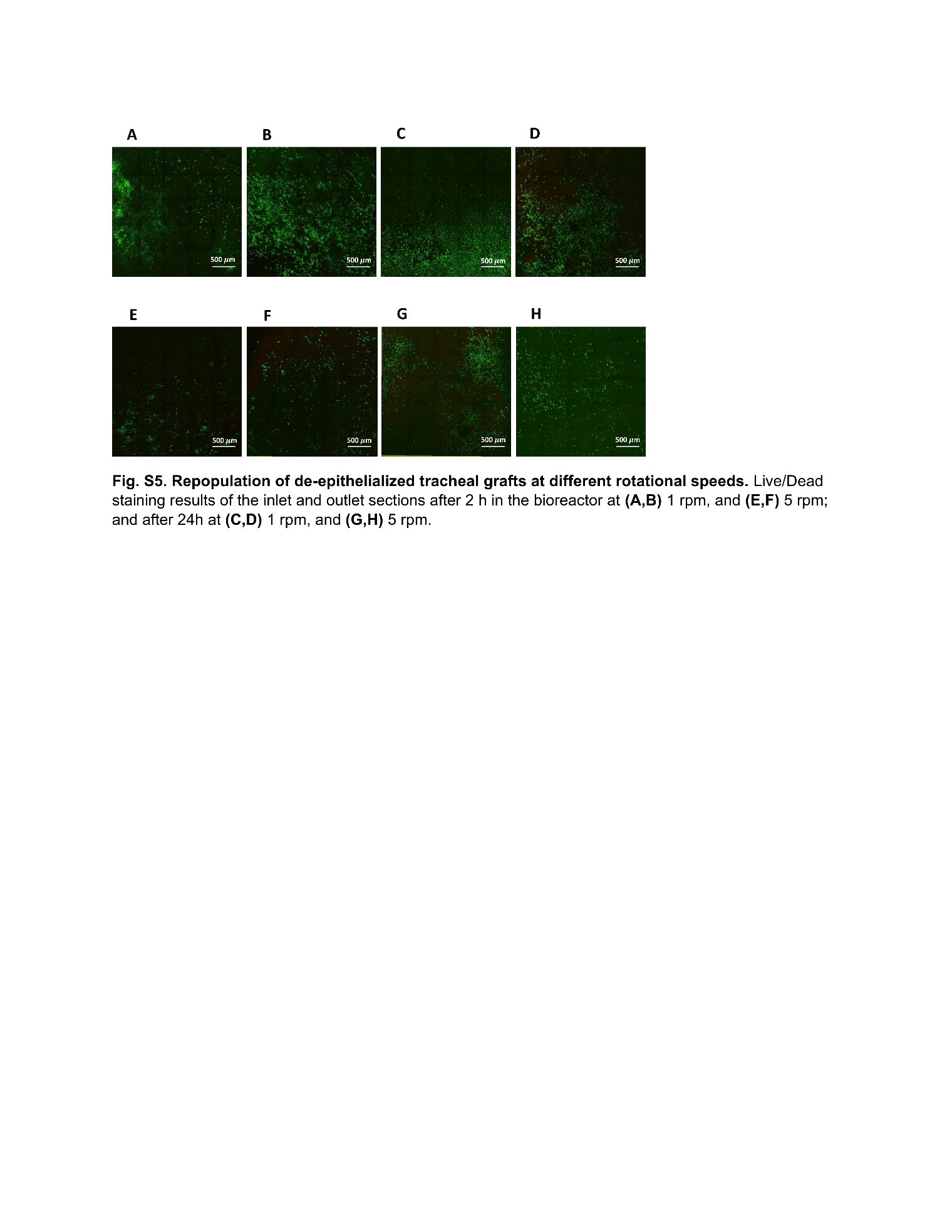


**Fig. S5. Repopulation of de-epithelialized tracheal grafts at different rotational speeds.** Live/Dead staining results of the inlet and outlet sections after 2 h in the bioreactor at **(A,B)** 1 rpm, and **(E,F)** 5 rpm; and after 24 h at **(C,D)** 1 rpm, and **(G,H)** 5 rpm

.
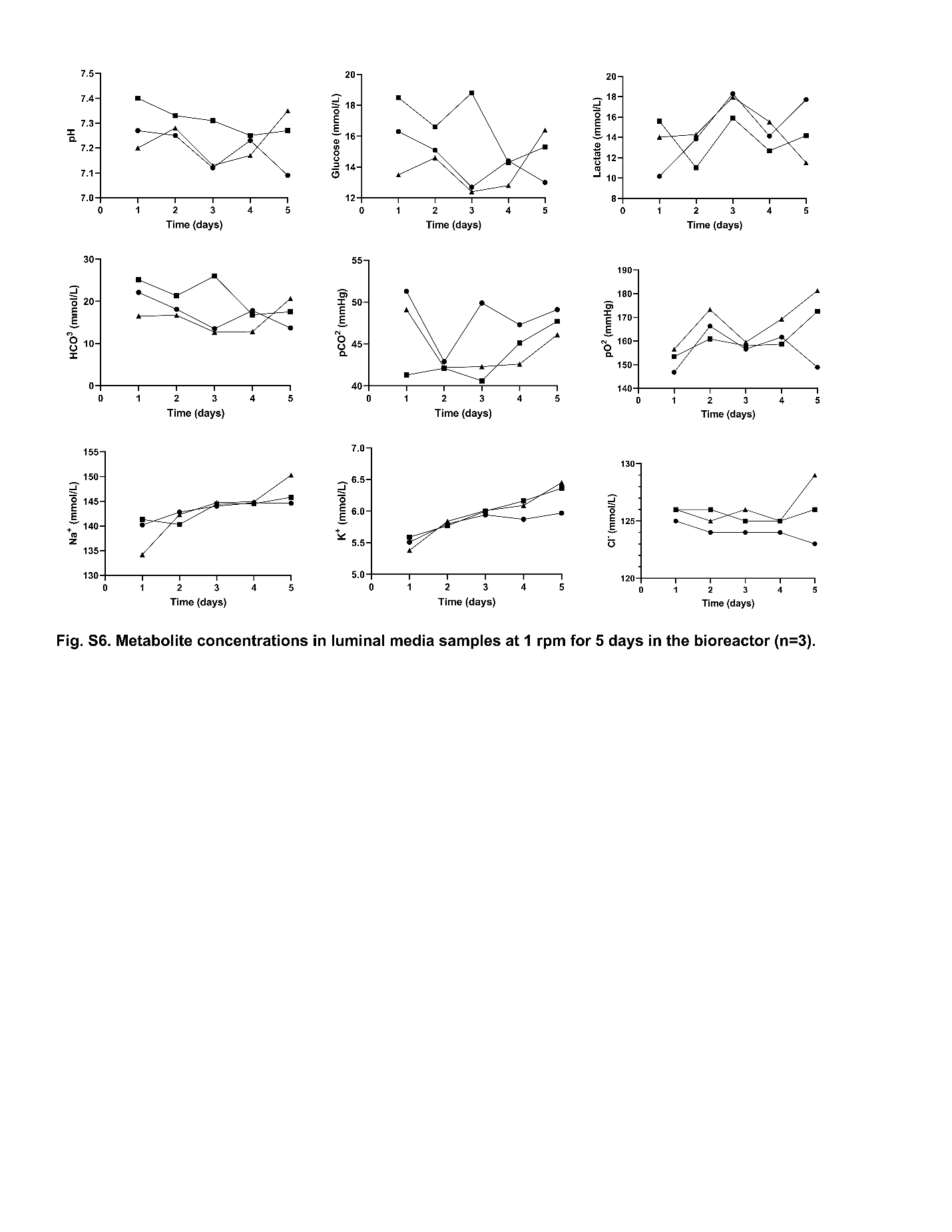


**Fig. S6. Metabolite concentrations in luminal media samples at 1 rpm for 5 days in the bioreactor (n=3)**

**.**
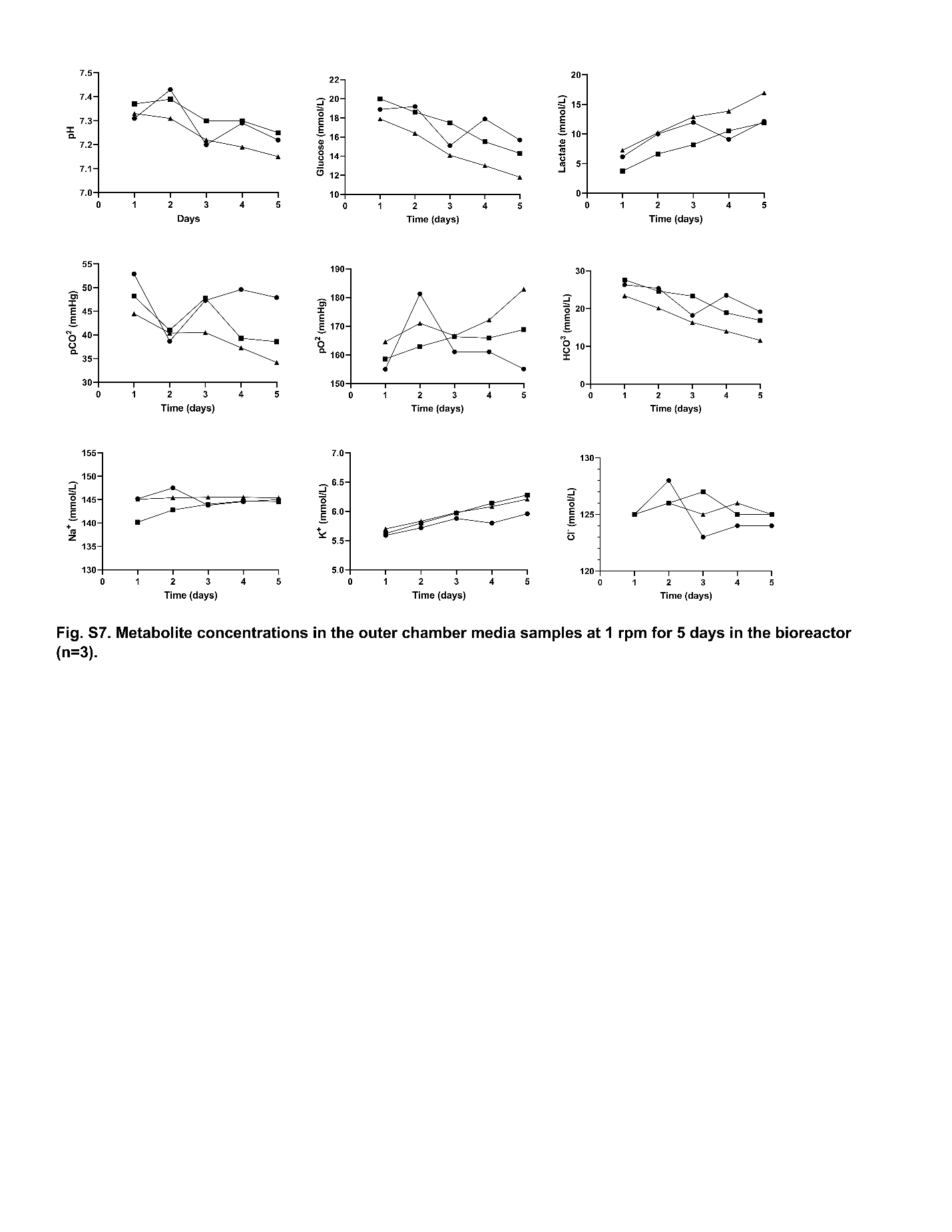


**Fig. S7. Metabolite concentrations in outer chamber media samples at 1 rpm for 5 days in the bioreactor (n=3).**
